# Clinical criteria for anaphylaxis: Comparing apples and pears

**DOI:** 10.1016/j.waojou.2023.100798

**Published:** 2023-07-12

**Authors:** Paul J. Turner, Victoria Cardona

**Affiliations:** a National Heart & Lung Institute, Imperial College London, London, UK; b Allergy Section, Department of Internal Medicine, Hospital Vall d'Hebron, ARADyAL Research Network, Barcelona, Spain

We read with interest the recent analysis by Çolak et al publishing in World Allergy Organization Journal,^1^ in which the authors reported that 6 of 204 patients with anaphylaxis according to National Institute of Allergy and Infectious Diseases/Food Allergy and Anaphylaxis Network (NIAID/FAAN) criteria^2^ did not meet the World Allergy Organization (WAO) 2020 criteria for anaphylaxis.^3^

Unfortunately, the authors do not provide sufficient information on these 6 cases for the reader to make an assessment as to whether the reaction descriptions constitute anaphylaxis or not.

Anaphylaxis is defined by WAO as “Anaphylaxis is a serious systemic hypersensitivity reaction that is usually rapid in onset and may cause death.”^2^ There is no gold-standard definition for anaphylaxis, which is a clinical diagnosis. Clinical criteria are a diagnostic aid, not a gold-standard definition.^2^, ^3^, ^4^ One reason for the development of the new WAO anaphylaxis criteria was to try and solve some of the ambiguity of the NIAID/FAAN criteria which can lead to non-anaphylaxis reactions (such as skin and mild gastrointestinal symptoms) being wrongly diagnosed as anaphylaxis.^3^^,^^4^

Çolak et al inform us that “none of these 6 patients [had] skin involvement but [had] findings related to 2 systems other than hypotension or bronchospasm or larynx edema.” If these individuals had cardiovascular or respiratory compromise, then this would have been captured by the second criterion in the WAO anaphylaxis guidance of 2020. Therefore, the implication is that these 6 individuals must have had non-severe gastrointestinal features without skin involvement but with subjective respiratory symptoms (chest tightness?) without objective respiratory compromise. Arguably, such reactions would not constitute anaphylaxis. Alternatively, some cases may have involved a vasovagal event, which again is not anaphylaxis. The lack of detail does not, unfortunately, support the author's assertion that “WAO diagnostic criteria seem to be insufficient in some patients.” The fact that 3 of the 6 individuals has skin sensitisation to the trigger agent does not support a diagnosis of anaphylaxis, but rather an allergic reaction independent of severity.

We urge the authors to provide more clarity over these 6 cases, to allow the reader to properly evaluate the cases and decide whether the authors’ conclusion is justified.
